# Why Is It So Hard to Find Persistent Borreliella burgdorferi?

**DOI:** 10.1128/mbio.02020-22

**Published:** 2022-08-22

**Authors:** Steven W. Luger

**Affiliations:** a The Lyme Disease Expert, West Hartford, Connecticut, USA; University of Texas Health Science Center at Houston

**Keywords:** *Borreliella burgdorferi*, Lyme disease, counting, infection

## LETTER

As a clinician since the early days of Lyme disease, I was eager to read the important article, “Borreliella burgdorferi antimicrobial-tolerant persistence in Lyme disease and posttreatment Lyme disease syndromes,” by F. C. Cabello et al. (1). Though most of the microbiology in the article was far beyond my comprehension, there are several clinical issues that I would like to address.

First, the author’s introductory comment that the number of cases of Lyme disease each year in the United States is “estimated to be at least 476,000 cases…” The CDC website cites 2 vastly different Lyme disease counts. The first estimate is 35,000 cases per year, based on the case reports from the Health Departments of each state, while “…estimates using other methods suggest that approximately 476,000 people may get Lyme disease each year” (2). The figure of 476,000 cases per year is based on insurance claims. Insurance claims vastly overestimate the number of cases of Lyme disease, as they are based on ICD-9 codes submitted by practitioners so insurance will cover the cost of a test. Patient no. 1 had a tick bite and 3 weeks later was feeling tired; a Lyme enzyme immunoassay (EIA) is ordered with a diagnosis of “Lyme disease.” The test comes back negative but is already reported in insurance statistics. Patient no. 2 has an erythema migrans (EM) rash of Lyme disease; his provider orders a Lyme EIA, which is reported as a case of Lyme disease by the insurance company; 1 month later, the patient is fatigued and another Lyme EIA is ordered and is again submitted as an insurance claim, with a diagnosis as a Lyme disease case. (The insurance has no way of knowing who has or does not have positive serology). Still feeling tired, the patient sees an infectious disease (ID) doctor who orders Lyme serology and a Western blot. This again is reported by the insurance company as another Lyme case—1 patient reported 3 times. The true number is likely >30,000 but clearly <470,000.

A few important clinical issues were not addressed. Lyme disease has existed in Europe for >100 years and for >50 years in the United States, yet we did not see a large population of patients with the late ravages of Lyme disease. This is likely because either the human host or our immune system makes ongoing infection rare in an inhospitable environment. Secondly, despite a tick infection rate of 40 to 50% in endemic areas of Connecticut, New York, and New Jersey, the vast majority of tick bites don’t result in the victim getting Lyme disease. Why is the infection rate not approaching 40%? Finally, in the patients that I have seen with posttreatment Lyme disease (PTLD), their Western blots generally do not show the expansion in the number of bands that one would expect if there were viable B. burgdorferi-expressing proteins that the host’s B cells are recognizing.

With such a large number of Lyme disease cases/year in the United States, one would think that finding “persisting spirochetes” would not be so rare. Could the reason be that “persisting spirochetes” are almost never present?

## References

[1] Cabello FC, Embers ME, Newman SA, Godfrey HP. 2022. *Borreliella burgdorferi* antimicrobial-tolerant persistence in Lyme disease and posttreatment Lyme disease syndromes. mBio 13:e03440-21. doi:10.1128/mbio.03440-21. PMC9239140 35467428

[2] Centers for Disease Control and Prevention. 2021. Lyme disease. Centers for Disease Control and Prevention, Atlanta, GA. https://www.cdc.gov/lyme/stats/humancases.html.

